# ECG-Gated 4D-CTA Assessment of Intracranial Aneurysm Wall Dynamics and Longitudinal Size Change: An Exploratory Study

**DOI:** 10.3390/neurolint18050081

**Published:** 2026-04-27

**Authors:** Peter Jankovič, Kamil J. Chodzyński, Axel E. Vanrossomme, Karim Zouaoui Boudjeltia, Andrej Šteňo, Christian R. Wirtz, Ján Šulaj, Andrej Paľa

**Affiliations:** 1 Department of Neurosurgery, F.D. Roosevelt University Hospital with Policlinic, 975 17 Banska Bystrica, Slovakia; jsulaj@nspbb.sk; 2 Laboratory of Experimental Medicine (ULB 222 Unit), Free University of Brussels, CHU de Charleroi, 6110 Montigny-le-Tilleul, Belgium; kamil.chodzynski@humani.be (K.J.C.); karim.zouaoui@humani.be (K.Z.B.); 3 Medical Imaging Unit, Centre Hospitalier Universitaire de Charleroi, 6042 Charleroi, Belgium; axel.vanrossomme@humani.be; 4 Clinical Neuroscience Research Unit, Department of Neurosurgery, Faculty of Medicine, Comenius University, University Hospital Bratislava, 833 05 Bratislava, Slovakia; andrej.steno@fmed.uniba.sk; 5 Department of Neurosurgery, University of Ulm, Lindenallee 2, 89312 Günzburg, Germany; rainer.wirtz@bkh-guenzburg.de (C.R.W.); andrej.pala@uni-ulm.de (A.P.)

**Keywords:** spatial aneurysm wall pulsation, global volumetric aneurysm pulsation, ECG-gated 4D-CT angiography, aneurysm wall deformability

## Abstract

Background: The risk stratification of unruptured intracranial aneurysms (UIAs) relies largely on static clinical and morphological parameters, which may not fully capture aneurysm-specific wall behavior. ECG-gated four-dimensional computed tomography angiography (4D-CTA) enables the time-resolved assessment of aneurysm wall motion, but reliable interpretation requires the differentiation of biological motion from measurement uncertainty. Methods: In this prospective exploratory pilot study, ECG-gated 4D-CTA was used to evaluate the longitudinal aneurysm size change, global volumetric pulsation (GVP), spatial wall pulsation (SWP), intrinsic wall deformability and variability. Size change and pulsation were defined using predefined resolution- and noise-based thresholds. Spatial wall motion was assessed using phase-resolved three-dimensional displacement maps. Harmonic modeling isolated periodic pulsation, and residual variability exceeding empirically derived uncertainty limits was conservatively interpreted as deformability. Associations with aneurysm growth and ELAPSS scores were analyzed using exploratory statistics. Results: Eleven UIAs in ten patients were followed for 4.3 ± 1.1 years. A longitudinal size change occurred in six aneurysms (54.5%). Baseline GVP was present in eight aneurysms (73%) and SWP in nine (82%). GVP was not associated with a size change (*p* = 1.00). All aneurysms with a size change exhibited baseline SWP, whereas no size change was observed in aneurysms without SWP; however, this association did not reach statistical significance in this small exploratory cohort (*p* = 0.18). Conservative variability metrics were not associated with growth but correlated with baseline shape irregularity, particularly the undulation index (Spearman’s ρ up to ~0.90). Conclusions: In this small exploratory pilot cohort, spatial wall pulsation showed a descriptive directional pattern with longitudinal aneurysm size changes, whereas global volumetric pulsation did not. These findings are preliminary, should be interpreted cautiously, and require confirmation in larger, adequately powered longitudinal studies before clinical application.

## 1. Introduction

Intracranial aneurysm rupture is a devastating cause of subarachnoid hemorrhage, yet identifying which unruptured aneurysms will progress or rupture remains a major challenge in cerebrovascular medicine [1,2,3].

Intracranial aneurysms affect approximately 3–5% of the adult population [1,4,5]. Although many remain asymptomatic, rupture leads to subarachnoid hemorrhage, a life-threatening condition associated with high mortality and long-term neurological disability [2,6]. Clinical management therefore depends on estimating the likelihood of aneurysm growth or rupture in order to balance the benefits of preventive treatment against its procedural risks [3,7].

Current risk stratification relies primarily on static anatomical and clinical factors such as the aneurysm size, location, and patient-related characteristics [7]. ECG-gated 4D-CTA adds a dynamic dimension to intracranial aneurysm assessment beyond static morphology. Early studies focused on pulsating blebs as potential markers of local wall weakness. Kato first applied 4D-CTA in 15 unruptured aneurysms, with pulsation detected in 10/15 cases [8]. Hayakawa then reported pulsation in 4/23 ruptured aneurysms, with surgical correspondence to the rupture site in two, and Ishida identified pulsating blebs in 9/28 saccular aneurysms, again with confirmation of the rupture point in two operated ruptured aneurysms [9,10]. These studies established the biological relevance of focal spatial wall pulsation, but they also prompted early concern about validity: in the 2006 Matsumoto/Ishida correspondence, pulsatile changes on 4D-CTA were explicitly divided into artifact-related motion and true aneurysm wall pulsation, with a call for further quantitative validation [11].

A further methodological step was the quantification of local wall motion. Karmonik et al. reported average wall displacement of 0.15 mm (range 0.04–0.31 mm) and showed that aneurysm motion relative to the vascular tree can be separated from motion of the aneurysm as a whole, emphasizing the heterogeneous and localized nature of wall behavior [12]. Later, 4D-CTA studies shifted toward global volumetric pulsation: Kuroda et al. found mean cardiac cycle-related volume changes of 5.40% ± 4.17% in aneurysms versus 4.20% ± 2.04% in normal arteries, while Dissaux et al. reported a mean volume variation of 10.9% (95% CI 4–17%) and concluded that volume is the most reproducible dynamic parameter, especially in aneurysms larger than 5 mm [13,14].

At the same time, the field has increasingly recognized that the major issue is not only detecting pulsation but distinguishing biological motion from measurement uncertainty. A systematic review by Stam et al. showed marked methodological heterogeneity and reported relative volume changes ranging from 5% to 36%, concluding that the true magnitude of aneurysm pulsation remains uncertain [15]. Most recently, Xie et al. introduced the first 4D-CTA-based quantification of irregular pulsation using surface displacement and strain analysis and showed that the stepwise first principal strain was higher in aneurysms with irregular pulsation than in those without (0.20 ± 0.01 vs. 0.16 ± 0.02; *p* = 0.033); importantly, they also identified measurement uncertainty as a key unresolved issue [16].

In this context, the present study integrates the two principal dynamic perspectives that have emerged in the literature yet are seldom assessed together: global volumetric pulsation, which reflects the overall cyclic behavior of the aneurysm, and spatial wall pulsation, which reveals focal heterogeneity and potentially vulnerable wall regions [8,9,10,11,12,13,14,15,16]. However, the relative contributions of periodic pulsation, intrinsic wall deformability, variability, and measurement uncertainty to the observed wall motion remain poorly understood [11,15,16].

Therefore, the primary aim of this exploratory longitudinal pilot study was to determine whether ECG-gated 4D-CTA can detect biologically meaningful aneurysm wall dynamics beyond static morphology by jointly assessing global volumetric pulsation and spatial wall pulsation within a conservative, uncertainty-aware analytical framework. Secondary aims were to distinguish periodic pulsation from non-periodic residual wall behavior, to explore the relationships of these dynamic parameters with longitudinal aneurysm size changes, and to assess whether spatially resolved wall pulsation provides complementary information to established clinical and morphological risk indicators in identifying aneurysms prone to structural remodeling.

## 2. Materials and Methods

### 2.1. Study Population

We conducted a single-center, prospective, exploratory pilot study evaluating aneurysm size changes and cardiac cycle-related wall motion using ECG-gated four-dimensional CT angiography (4D-CTA). The cohort comprised ten adult patients with eleven unruptured saccular intracranial aneurysms that had been previously diagnosed on CTA or MRA. Although preventive endovascular or microsurgical treatment was recommended following multidisciplinary evaluation, all patients opted for surveillance and agreed to dynamic CT follow-up. Baseline and follow-up ECG-gated 4D-CTA were performed with a minimum interval of one year.

Inclusion criteria were (i) age 18–80 years; (ii) unruptured saccular intracranial aneurysm ≥3 mm; (iii) ability to provide written informed consent; and (iv) ECG-gated 4D-CTA examinations of sufficient quality for segmentation and phase-resolved analysis.

Exclusion criteria were (1) age < 18 or >80 years; (2) contraindication to iodinated contrast agents; (3) impaired kidney function; (4) pregnancy; (5) inability to provide informed consent; (6) fusiform, dissecting, or mycotic aneurysms; (7) symptomatic or ruptured aneurysms; (8) aneurysm size < 3 mm; and (9) insufficient image quality due to motion artifacts; (10) a complex (adherent) anatomical situation between the sac and surrounding vessels.

After applying all criteria, we obtained a cohort of ten patients with 11 aneurysms. The most important clinical and demographic characteristics are summarized in Table 1.

**Figure 1:**
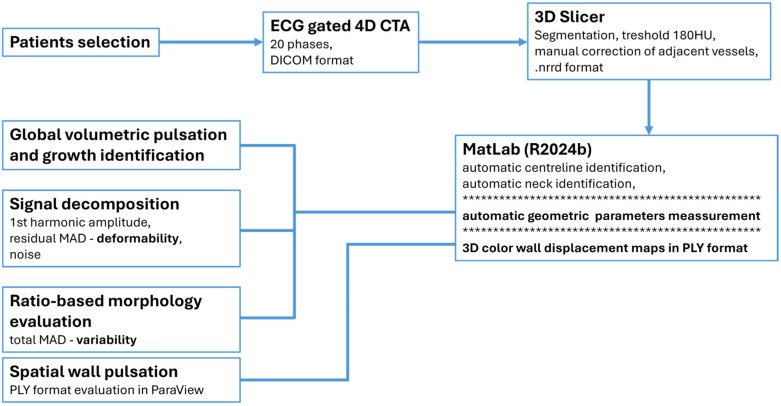
Workflow diagram of methodological concept. The MATLAB block represents the core processing stage, automatic generating outputs for final evaluation. Asterisks delineate three distinct but interrelated key tasks required for the final analysis.

The study was approved by the institutional ethics committee (protocol 33/2018). All participants provided written informed consent for study participation and for each CT examination. Data were anonymized prior to analysis. No a priori sample size calculation was performed because the study was designed as an exploratory pilot cohort study. The methodological workflow is described in the diagram below (Figure 1).

### 2.2. ECG-Gated 4D-CTA Acquisition

All examinations were acquired on a 320-detector-row CT system (Aquilion One, Toshiba, Japan) without changes in hardware or software configuration during the study. Acquisition parameters were as follows: tube voltage 130 kV; tube current 230 mA with automatic dose modulation; gantry rotation time 450 ms; in-plane spatial resolution 0.5 × 0.5 mm; and z-coverage 160 mm. Scanning was performed over a single cardiac cycle, with retrospective ECG gating covering 0% to 95% of the R-R interval in 5% steps (20 phases).

Bolus tracking was performed at the level of the C1 vertebra in the internal carotid artery, using a threshold of 180 HU. Patients received breathing instructions prior to acquisition to minimize respiratory motion. Contrast administration consisted of 50 mL iodinated contrast (weight-adjusted) injected at 5 mL/s, followed by a 40 mL saline flush. Data were stored in DICOM format. Each ECG-gated acquisition was reconstructed into 20 cardiac phases.

### 2.3. Image Segmentation, Automatic Neck Identification, and Generation of 3D Color Wall Displacement Maps

The CT DICOM files were preprocessed using 3D Slicer (v5.6.2) [17]. Before segmentation, the region of interest containing the aneurysm and parent arteries was isolated to reduce the computational burden. The grey-level threshold method with a fixed threshold of 180 HU was then used to separate the aneurysm and parent arteries from the background. All image segmentation preprocessing was performed by a single experienced neurosurgeon with more than 10 years of cerebrovascular experience. To reduce segmentation ambiguity, only aneurysms with a relatively simple adjacent vascular anatomy were included. For example, owing to uneven contrast distribution, small, attached vessels or irregular adjacent structures near the aneurysm sac may have appeared, which could have influenced the measured aneurysm volume. Cases with extensive adherent contact between the aneurysm sac and surrounding vessels were excluded, whereas limited close apposition was accepted when reliable delineation of aneurysm boundaries remained feasible. Therefore, manual correction was performed, when necessary, with the aim of keeping such intervention to a minimum [18,19]. The final segmented volume of the aneurysm and surrounding parent arteries was saved as a binary .nrrd file. A standardized workflow was applied throughout. Intraobserver and interobserver variability were not formally evaluated in this pilot study [20].

The .nrrd files for each 20-phase interval were loaded into MATLAB (v2024b), where automated evaluation was performed. To smooth the surface of the volume, a weighted moving-average filter with a box window of size [3, 3, 3] was used. The volumes were also resampled five times using linear interpolation. Subsequently, the centerlines and key points, such as endpoints and branch points, were extracted. The centerline points were then divided into branch centerline points and aneurysm points. For each branch, the centerline points were projected onto the aneurysm surface in the direction of the aneurysm sac point. The resulting surface points were then used to compute the distance to the aneurysm branch point. The minimum distance indicated the cut point between the branch and the aneurysm sac. If there were more than two branches, the cut plane was calculated based on the three points closest to the aneurysm neck point. Otherwise, the cut plane was constructed using the two identified branch points and the perpendicular vector between the highest surface point of the aneurysm and the line defined by the two branch points.

This plane was then used to separate the aneurysm from the parent arteries. Finally, the aneurysm sac was subjected to geometric analysis.

The isosurfaces were calculated from the resulting volumes using a MATLAB (v2024b) function to create 3D color maps of wall displacement. From these isosurfaces, the vertices were extracted. Movement was calculated as the Euclidean magnitude, representing the total change and always remaining positive [12,16].

$$d_{i,t}=\sqrt{\sum_{j=1}^{3}{\left({v(t)}_{i,j}-r_{j}\right)}^{2}}$$

where ${v(t)}_{i,j}$ is the *j* th coordinate (x, y, or z) of the *i*th neighbor vertex at time *t*, $r_{j}$ is the *j*th coordinate of the reference vertex, and d*_i_*(*t*) is the resulting distance for the *i*th vertex at time *t*.

### 2.4. Geometric Analysis of the Aneurysm

Automated geometric quantification was performed using in-house MATLAB software (R2024b). After centerline extraction and the automatic identification of a planar neck, as described above, we computed the following.

Direct geometric parameters: height (H), maximum height (H_max_), maximum size (L_max_), sac volume (V_sac_), sac-plus-parent-artery volume (V_sacpar_), sac surface area (S_sac_), sac-plus-parent-artery surface area (S_sacpar_), maximum neck diameter (N_max_), neck perimeter (N_perimet_), and neck area (N_area_) [21,22].

Ratio-based morphology indices: aspect ratio (AR), size ratio (SR), undulation index (UI), non-sphericity index (NSI), bottleneck factor (BF), and conicity parameter (CP) [22,23].

Definitions of all parameters are provided in Supplementary Table S1.

### 2.5. Assessment of Aneurysm Size Change

The aneurysm size change was evaluated using changes in the mean sac volume between baseline and follow-up. The change was considered significant if

$$|V_{mean2}-V_{mean1}|>2\times \sqrt{SD_{1}^{2}+SD_{2}^{2}}$$

where $V_{mean1}$ and $V_{mean2}$ denote the mean sac volumes across 20 phases at baseline and follow-up, respectively, and $SD_{1}$ and $SD_{2}$ denote the corresponding phase-wise standard deviations.

### 2.6. Global Volumetric Pulsation (GVP)

Global volumetric pulsation (GVP) was defined as a sac volume change over the cardiac cycle. To avoid classifying noise as pulsation, pulsation was considered present only if both of the following criteria were met:

1. The resolution-limited minimum detectable volume change exceeded 3 mm^3^ [24,25];
2. The measured pulsation amplitude exceeded a noise-based threshold of $3\sigma_{noise}$.

**Figure 2:**
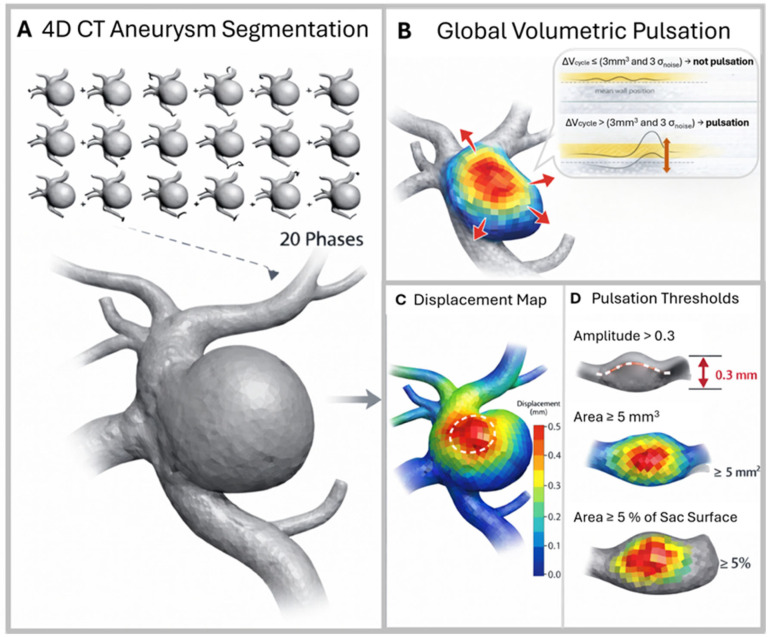
Four-dimensional CTA-based aneurysm segmentation and quantitative pulsation analysis. (**A**) The geometry of the aneurysm is segmented from 4D-CT angiography across 20 cardiac phases. (**B**) Global volumetric pulsation is defined as a volume change (ΔV) that exceeds the noise threshold and the minimum detectable volume of 3 mm^3^ (yellow field over the wall—red arrow). (**C**) A 3D wall displacement map shows the spatial distribution of wall motion (mm). (**D**) Pulsation is defined using the following thresholds: displacement amplitude > 0.3 mm, pulsating area ≥ 5 mm^2^, and ≥5% of the aneurysm sac surface.

The noise standard deviation was estimated robustly from successive inter-phase differences:

$$d_{i}=V_{i+1}-V_{i}\;\;\;\;\;(i=1\ldots 19)$$

$$\sigma_{noise}=\frac{MAD(d_{i})}{0.6745\sqrt{2}},$$

where MAD denotes the median absolute deviation. Division by 0.6745 converts the MAD to the Gaussian-equivalent standard deviation, and division by $\sqrt{2}$ corrects for the doubling of variance in first differences of independent equal-variance noise terms. This procedure was used only to estimate the noise level for thresholding; no denoising or noise subtraction was applied to the GVP curve. The $3\sigma_{\mathrm{noise}}$ threshold corresponds to a conventional Gaussian-equivalent three-sigma criterion (Figure 2) [26,27].

### 2.7. Spatial Wall Pulsation (SWP) from Surface Displacement Maps

Because a stable volume does not exclude focal shape changes, local wall motion was assessed using 3D wall displacement mapping across the 20 cardiac phases [12,16,25]. Aneurysm surface displacement amplitudes were computed using an in-house MATLAB (v2024b) script and visualised as displacement maps (0 to ≥0.5 mm). Wall displacement extraction and visualisation were performed in sofware ParaView (v6.0.1).

Given that aneurysm wall motion may be at or below the voxel size, we applied conservative spatial coherence criteria to define detectable SWP [25]. SWP was considered present only if a region exceeded the following thresholds (Figure 2): displacement amplitude > 0.3 mm, affected surface area ≥ 5 mm^2^, and ≥5% of the total sac surface area (Figure 2) [28].

SWP was classified as follows:

Focal SWP: < 20% of total sac surface area involved;

Heterogeneous SWP: ≥ 20% distinct pulsating regions;

No detectable SWP: < 5% thresholds not exceeded.

Pulsation metrics (GVP and SWP) were evaluated in relation to the ELAPSS score [29].

### 2.8. Processing and Reliability Assessment of Phase-Resolved Geometric Signals

ECG-gated 4D-CTA measurements are influenced by phase-dependent noise, segmentation uncertainty, and the limited spatial resolution. We therefore applied a two-step reliability-based approach.

#### 2.8.1. Dynamic Signal Qualification (Quality Control)

For each aneurysm and time point (baseline and follow-up), phase-resolved signals (20 phases) were summarized via the minimum, maximum, mean, median, signal range $\Delta X=X_{max}-X_{min}$, robust noise metrics (*SD_noise_* and MAD), and signal-to-noise ratio (*SNR*), defined as

$$SNR=\frac{\Delta X}{SD_{noise}}$$

Signals meeting predefined variability criteria and exceeding the minimum SNR threshold were classified as dynamically valid for further interpretation.

#### 2.8.2. Decomposition of Pulsation and Reliability-Based Deformability Estimation

Phase-resolved geometric signals reflect (i) periodic pulsation, (ii) potential non-periodic deformation, and (iii) measurement uncertainty. To isolate the periodic component, we fitted a first-harmonic model to each signal; the pulsation amplitude was defined as the amplitude of the first harmonic [30,31]. The fitted periodic component was subtracted from the measured signal to obtain residuals.

Residual variability was quantified using the residual MAD [26,27]. Because residuals contain both noise and true non-periodic motion, the residual MAD was interpreted conservatively as deformability only if it exceeded empirically derived uncertainty bounds.

**Figure 3:**
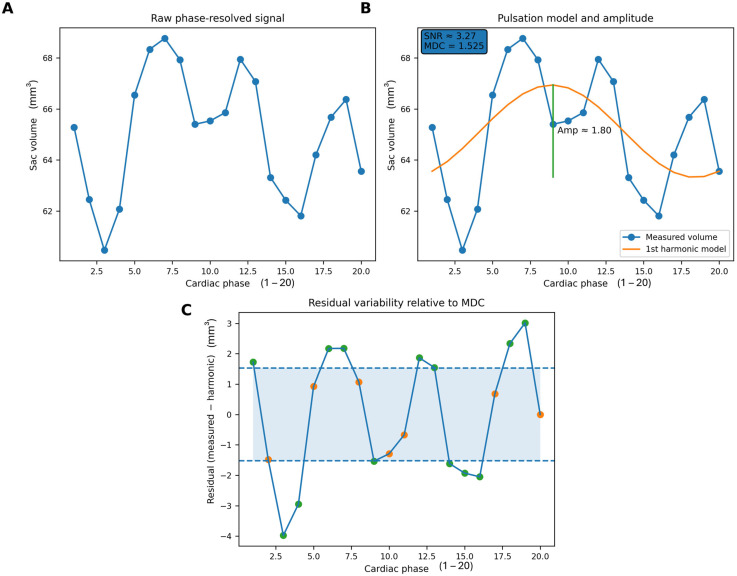
Decomposition of phase-resolved aneurysm volume signal and detection of non-periodic variability. (**A**) Raw aneurysm sac volume measured across 20 cardiac phases using ECG-gated 4D-CTA. (**B**) First-harmonic modeling isolates the periodic pulsation component (orange curve); the vertical bracket (green line) indicates the pulsation amplitude, while the inset reports the signal-to-noise ratio (SNR) and the minimum detectable change (MDC) derived from repeatability analysis. (**C**) Residual variability after subtraction of the harmonic component. The shaded region represents the MDC interval, within which variability is indistinguishable from measurement uncertainty (orange dots), whereas points exceeding this interval indicate detectable non-periodic wall motion (green dots).

Measurement repeatability and the minimum detectable change (MDC) were characterized using Bland–Altman analysis [32,33]. Residual MAD values not exceeding the noise floor/MDC were considered indistinguishable from measurement uncertainty and were not interpreted (Figure 3) [33,34,35]. Accordingly, deformability in this study represents a lower-bound estimate of intrinsic non-periodic wall behavior rather than a noise-free mechanical property. The absence of deformability values indicates intentional exclusion by reliability criteria rather than missing data. The detailed mathematical formulation of the signal decomposition, including harmonic modeling, residual MAD estimation, and Bland–Altman-derived minimum detectable change, is provided in Appendix A.

After signal decomposition, deformability was derived as a quantitative descriptor of aneurysm wall motion. Given the exploratory design and limited cohort size, all deformability analyses were considered hypothesis-generating. Specifically, we assessed (i) the mechanical coupling between the pulsation amplitude and deformability, (ii) the temporal stability of the deformability metric, (iii) the influence of the aneurysm geometry on pulsatile motion, and (iv) the associations between deformability and aneurysm growth using the conservative deformability definition. Because strict quality control and minimum detectable change criteria were applied, the effective sample size for several deformability analyses was limited; these analyses were therefore intended to evaluate methodological feasibility and exploratory signal patterns rather than to support definitive inferential conclusions.

#### 2.8.3. Morphology-Based Correlation Analysis

Ratio-based morphology indices were analyzed as static descriptors [21,22]. Dynamic hypotheses were evaluated using direct geometric measurements to avoid non-linear error propagation inherent to ratios [36]. As ratio-based parameters inherently normalize the scale, the residual MAD was not applied. For geometric parameters, the total MAD was used to characterize overall variability.

The baseline ratio-based morphology was explored for its correlation with the baseline variability (total MAD), pulsation amplitude, follow-up variability, longitudinal change in variability, and aneurysm size change.

In practical terms, pulsation reflects the regular heartbeat-driven expansion and contraction of the aneurysm; variability describes the overall magnitude of geometric change observed in the signal; and deformability represents additional non-periodic wall motion that remains after removing the pulsation component and exceeds the expected measurement uncertainty.

### 2.9. Statistics

All analyses were performed at the aneurysm level. Given the small sample size (11 aneurysms) and limited number of size change events (*n* = 6), analyses were predefined as exploratory and hypothesis-generating, and multivariable modeling was not performed so as to avoid overfitting.

Continuous variables were analyzed on their original continuous scales. Their distribution was assessed using the Shapiro–Wilk test. As several variables were not normally distributed and the study cohort was small, nonparametric methods were applied preferentially. Accordingly, associations between continuous variables were evaluated using Spearman’s rank correlation coefficient, while between-group comparisons were performed using the Mann–Whitney U test. Categorical variables were compared using Fisher’s exact test.

The primary analysis evaluated the association between SWP (detectable vs. not detectable) and aneurysm size changes (yes/no) using Fisher’s exact test, reporting odds ratios with exact 95% confidence intervals. Secondary analyses included Spearman correlations (ρ) between ELAPSS and imaging/morphologic parameters and Mann–Whitney U tests for group comparisons. Variable sample sizes were expected because deformability metrics were intentionally filtered by quality control and MDC criteria; the sample size is reported for each comparison. For several deformability metrics, fewer than five paired baseline–follow-up observations remained after filtering; consequently, these analyses were interpreted descriptively and as hypothesis-generating rather than as a basis for robust inferential testing.

All tests were two-sided with α = 0.05. Given the exploratory nature of the study and the limited sample size, the analyses were interpreted primarily in terms of the effect direction and magnitude rather than formal statistical significance.

No adjustment for multiple comparisons was applied due to the exploratory design; emphasis was placed on effect sizes and confidence intervals. This should be considered when interpreting the secondary findings.

## 3. Results

### 3.1. Assessment of Aneurysm Size Change and Pulsation

#### 3.1.1. Study Population and Baseline Characteristics

Eleven unruptured saccular intracranial aneurysms in ten patients were included. The mean age was 63.1 ± 13.2 years (median 68, range 32–79). Baseline demographic and aneurysm characteristics are provided in Supplementary Table S2. The interval between baseline and follow-up imaging ranged from 2 to 6 years (mean 4.3 ± 1.1 years).

The baseline maximum aneurysm size averaged 6.26 mm (range, 3.49–9.80 mm), increasing to 6.44 mm (range, 3.87–10.38 mm) at follow-up. The mean aneurysm volume was 63.5 mm^3^ at baseline (range, 12.41–203.73 mm^3^) and 76.4 mm^3^ at follow-up (range, 14.26–220.85 mm^3^). The mean ELAPSS-predicted 3-year growth risk was 17.8 ± 9.6 (range 7.8–42.7) (Table 2).

At baseline, global volumetric pulsation (GVP) was detected in 8/11 aneurysms (73%) and spatial wall pulsation (SWP) was detected in 9/11 aneurysms (82%), including five focal and four heterogeneous patterns.

During follow-up, an aneurysm size change according to predefined volumetric criteria occurred in 6/11 aneurysms (54.5%): five showed enlargement and one showed a reduction.

#### 3.1.2. Relationship Between Global Volumetric and Spatial Wall Pulsation

Baseline GVP and SWP showed partial concordance.

Among aneurysms with detectable GVP (*n* = 8), SWP was present in six cases (four heterogeneous, two focal), and no detectable SWP was observed in two cases.

Among aneurysms without GVP (*n* = 3), SWP was still detected in two cases (both heterogeneous), and one aneurysm had no detectable pulsation.

#### 3.1.3. Association Between Pulsation and Aneurysm Size Change

All aneurysms demonstrating a size change exhibited detectable spatial wall pulsation at baseline (6/6), whereas no aneurysm without SWP was enlarged (0/2). This corresponded to complete separation in the contingency table (6/9 vs. 0/2; Fisher’s exact *p* = 0.18), with an infinite conditional odds ratio and an exact 95% confidence interval extending from 0.24 to infinity; the absolute risk difference was 0.67 (95% CI, −0.06 to 0.88) (Table 3). These findings support a directional association between baseline SWP and subsequent aneurysm remodeling, whereas GVP did not demonstrate a comparable relationship in this exploratory dataset (Table 3). Given the small number of aneurysms and sparse cell counts, these estimates should be interpreted as hypothesis-generating.

These findings indicate that spatial wall displacement mapping identified focal wall motion in some aneurysms without measurable global volumetric pulsation. No consistent relationship between the baseline global volumetric pulsation and longitudinal aneurysm size change was observed in this small cohort (4/8 vs. 2/3; Fisher’s exact *p* = 1.00), corresponding to a conditional odds ratio of 0.53 (95% CI, 0.01 to 14.52) and an absolute risk difference of −0.17 (95% CI, −0.56 to 0.37).

#### 3.1.4. Integrated Analysis of ELAPSS Score, Pulsation, and Size Change

ELAPSS scores tended to be higher in aneurysms demonstrating a size change than in those without detected growth (19.1 ± 11.6 vs. 16.8 ± 5.6), although the difference was small and imprecise when expressed as a standardized effect size (Hedges’ g = 0.22, 95% CI, −0.87 to 1.31). Among aneurysms with an ELAPSS-predicted 3-year growth risk below 20%, a size change was observed only in those with detectable SWP, whereas no aneurysm without SWP was enlarged in this lower-risk subgroup (Table 4). Owing to the small subgroup size, this analysis remains exploratory and underpowered.

#### 3.1.5. Follow-Up Pulsation

At follow-up imaging, most aneurysms showed a reduction or loss of detectable GVP and SWP. Persistent pulsation was more common among aneurysms that had demonstrated a longitudinal size change. However, reduced pulsation was not uniformly associated with stability, and loss of pulsation occurred in both growing and stable aneurysms.

#### 3.1.6. Visual Presentation

**Figure 4:**
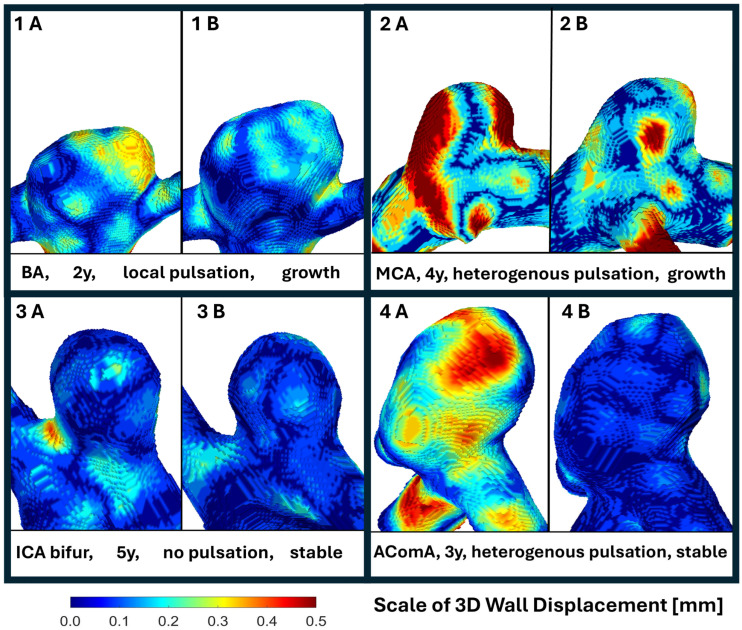
Representative 3D wall displacement maps of intracranial aneurysms derived from ECG-gated 4D-CTA. For each case (1–4), two views (**A**—baseline, **B**—follow-up) illustrate the spatial distribution of aneurysm wall motion during the cardiac cycle. Color maps represent the magnitude of 3D wall displacement (0–0.5 mm), with blue indicating minimal motion and yellow–red indicating higher displacement. Aneurysm 1 shows focal pulsation on the right side at baseline, followed by aneurysm growth and loss of pulsation after 2 years of follow-up. Aneurysm 2 demonstrates heterogeneous pulsation at baseline and stabilized after minor growth in 4 years. Aneurysm 3 remained stable without detectable pulsation during 5 years of follow-up. Aneurysm 4 exhibited heterogeneous pulsation at baseline and stabilized after 3 years.

Representative examples of spatial wall pulsation patterns and their longitudinal evolution are shown in Figure 4. Case 1 demonstrates focal spatial wall pulsation at baseline. The yellow region indicates the area of maximal wall displacement and corresponds to the location of subsequent aneurysm enlargement observed during follow-up. Case 2 shows heterogeneous pulsation involving multiple regions of the aneurysm surface, with minor aneurysm growth followed by stabilization; subtle morphological changes in the sac are visible. Case 3 exhibits no detectable pulsation and remained morphologically stable throughout follow-up. Case 4 demonstrates heterogeneous pulsation at baseline without a significant longitudinal size change. These examples illustrate the heterogeneous relationship between spatial wall pulsation patterns and subsequent aneurysm evolution. Baseline aneurysm wall pulsation appears to localize mechanically active regions of the aneurysm sac that may correspond to sites of subsequent morphological remodeling or growth during longitudinal follow-up. Displacement maps for the remaining aneurysms are provided in Appendix B.

### 3.2. Signal Qualification, Deformability Estimates, and Geometric Progression 

All geometric signals met the predefined dynamic qualification criteria.

After applying conservative reliability and minimum detectable change thresholds, deformability estimates were available in fewer than five paired baseline–follow-up aneurysms for several metrics, precluding a robust inferential analysis for most predefined deformability hypotheses.

The exploratory correlation analysis of pulsation progression versus geometric changes across all 11 aneurysms showed no statistically significant associations (all *p* > 0.1). The Spearman correlation coefficients ranged from weak to moderate in magnitude (|ρ| ≈ 0.07–0.49) and were inconsistent in direction.

The largest observed correlation coefficients in this exploratory dataset were as follows: ΔN_max_ (ρ = 0.473, *p* = 0.142), ΔHeight_max_ (ρ = −0.491, *p* = 0.125), ΔV_sacpar_ (ρ = 0.40, *p* = 0.223). None reached statistical significance (Table 5).

Overall, changes in pulsation amplitude did not demonstrate a consistent association with geometric progression in this cohort.

### 3.3. Associations Between Aneurysm Morphology and Geometric Variability

#### 3.3.1. Linear and Neck-Based Variability

Linear and neck-based variability metrics did not demonstrate significant associations with conventional morphological descriptors (AR, SR, BF, CP).

Across these domains, the undulation index (UI) showed the strongest observed relationships with the variability magnitude, while the non-sphericity index (NSI) showed weaker, trend-level associations.

No linear or neck-based variability metric was associated with longitudinal aneurysm size changes.

#### 3.3.2. Surface and Volume Variability

Surface- and volume-based variability metrics demonstrated consistent associations with baseline shape irregularity.

Baseline UI showed strong correlations with sac surface variability (S_sac_), sac-plus-parent-artery deformability (S_sacpar_), sac volume deformability (V_sac_), and combined volume deformability (V_sacpar_), with correlation coefficients of up to ρ ≈ 0.90 (*p* < 0.001) (Table 6). These associations indicate that a more irregular aneurysm morphology corresponds to higher measured residual variability.

The NSI demonstrated weaker, non-significant trend-level associations.

Other traditional morphological descriptors and size metrics showed no consistent relationship with the variability magnitude.

#### 3.3.3. Variability Versus Longitudinal Remodeling

Baseline variability metrics, whether linear, neck-based, surface, or volumetric, were not associated with aneurysm size changes (ΔVolume) or with longitudinal changes in variability.

Given the limited number of aneurysms meeting the strict variability reliability criteria, these findings should be interpreted cautiously.

## 4. Discussion

The main finding of the present exploratory pilot study is that spatially resolved aneurysm wall pulsation, rather than global volumetric pulsation, showed a clearer directional relationship with a subsequent longitudinal aneurysm size change. In contrast, neither baseline global volumetric pulsation nor baseline or progressive deformability demonstrated a consistent association with aneurysm remodeling during follow-up. A further important observation was that baseline aneurysm shape irregularity, particularly the undulation index, was the strongest determinant of geometric variability across cardiac phases, irrespective of the subsequent growth status. Taken together, these findings suggest that focal wall motion patterns derived from ECG-gated 4D-CTA may provide information that differs from both global dynamic metrics and static morphological descriptors. At the same time, the observed variability metrics appear to be influenced predominantly by baseline shape complexity rather than by longitudinal aneurysm progression itself. Given the small sample size and exploratory design, these results should be interpreted with caution and considered hypothesis-generating.

### 4.1. Spatial Wall Pulsation Versus Global Volumetric Pulsation

A central result of this study is the divergence between spatial wall pulsation (SWP) and global volumetric pulsation (GVP). At baseline, GVP was present in 8/11 aneurysms (73%) and SWP in 9/11 (82%), including five focal and four heterogeneous patterns. During follow-up, a size change occurred in 6/11 aneurysms (54.5%). GVP showed no clear relationship with longitudinal remodeling: growth or size changes occurred in 4/8 pulsating versus 2/3 non-pulsating aneurysms (*p* = 1.00, OR 0.53, 95% CI 0.01–14.52). In contrast, all aneurysms with a size change had baseline SWP (6/6), whereas no aneurysm without SWP was enlarged (0/2; *p* = 0.18, exact 95% CI 0.24 to infinity) (see Section 3). Although clearly exploratory, this directional contrast suggests that focal spatial displacement mapping may capture a biologically more informative signal than the whole-sac volume change.

A likely explanation is that GVP averages the behavior of the entire aneurysm sac and may therefore dilute localized deformation confined to a small wall region, whereas SWP specifically captures regional heterogeneity. This interpretation is consistent with earlier reports of focal bleb motion and with more recent studies linking irregular pulsation to an increased estimated rupture risk, small-aneurysm rupture, and larger dynamic morphologic changes during the cardiac cycle [8,9,10,16,37,38,39]. It is also consistent with the literature showing that dynamic wall motion is frequently local and non-uniform rather than globally distributed [10,12,15,16].

A size effect is also likely relevant. Dissaux et al. reported a mean volume variation of 10.9% (95% CI 4–17%) and concluded that volume variation appears most reproducible in aneurysms >5 mm [14]. In the review by Stam et al. [15], the absolute volume change increased with the aneurysm size, and the reported mean relative volume changes include 5.4 ± 4.1% in Kuroda et al., 8.0 ± 4.6% in Firouzian et al., and 10.9% in Dissaux et al. [13,14,15,40]. Thus, GVP is partly scale-dependent: larger aneurysms are more likely to generate measurable whole-sac volume excursions, whereas smaller aneurysms may still show focal wall displacement that is spatially meaningful but volumetrically modest [13,14,15,40].

The relation with ELAPSS also suggests that SWP may add information beyond conventional static growth risk stratification [29]. In our cohort, ELAPSS was only slightly higher in aneurysms with a size change than in stable aneurysms (19.1 ± 11.6 vs. 16.8 ± 5.6; Hedges’ g = 0.22, 95% CI −0.87 to 1.31) (see Section 3). More importantly, in aneurysms with an ELAPSS-predicted 3-year growth risk < 20%, a size change occurred only in those with detectable SWP (5/5; 100%) and not in aneurysms without SWP, whereas GVP was present in both growing (4/5; 80%) and stable (4/4; 100%) aneurysms in this subgroup (see Section 3). Although underpowered, this pattern supports the view that SWP may reflect aneurysm-specific wall behavior not fully captured by a score based on static variables.

The three-dimensional wall displacement maps in Appendix B support this interpretation. They demonstrate focal or heterogeneous pulsation despite a minimal or absent global volumetric change, showing that the aneurysm shape may vary during the cardiac cycle even when the total sac volume remains relatively stable. In several cases (A1, A7, A10), focal pulsation visually corresponded to regions where a later morphological change or size expansion was observed. This reinforces the limitation of whole-sac volumetric metrics in detecting regionally unstable wall behavior.

The predominance of reduced or absent detectable pulsation at follow-up should be interpreted cautiously. Because baseline and follow-up scans were acquired on the same 320-row CT platform and analyzed with the same segmentation and pulsation framework, this finding is unlikely to reflect an intentional protocol change alone. However, the SWP threshold lies close to current spatial resolution limits, so small differences in contrast opacification, cardiac cycle regularity, reconstruction, segmentation, or phase-dependent image noise may influence whether pulsation remains above the detectability threshold [15,25,41]. Loss of detectable pulsation was seen not only in enlarging aneurysms but also in one aneurysm that decreased in size, suggesting that this phenomenon cannot be explained by growth alone. A more plausible interpretation is that detectable pulsation depends on both limited sensitivity to very small wall motion amplitudes and interval changes in aneurysm biology, hemodynamics, geometry, or wall mechanical properties [15,25,41]. Thus, the attenuation or disappearance of pulsation should not be interpreted as evidence of biological stability per se, but rather as a hypothesis-generating observation that may reflect both technical detectability limits and time-dependent aneurysm remodeling [15,25,41].

### 4.2. Imaging Uncertainty and Conservative Deformability Assessment

A major strength of the present study is that aneurysm wall dynamics were approached first as a measurement problem and only then as a biological one. This is essential because in vivo aneurysm pulsation measurement still lacks a gold standard, and apparent wall motion may reflect a mixture of true deformation, image noise, reconstruction artifacts, contrast-related intensity changes, segmentation uncertainty, and registration errors [15,16,25,41]. Systematic reviews and validation studies consistently show that low-amplitude pulsation estimates are highly method-dependent, especially when the expected wall motion approaches the spatial resolution limit of current imaging techniques [15,25,41].

Within this context, our framework is deliberately conservative. By separating the dominant periodic component from residual variability and interpreting residual behavior only when it exceeds uncertainty bounds derived from the MAD and MDC, it reduces the risk of overinterpreting weak residual fluctuations as true wall mechanics [26,33,34]. This is stricter than in most earlier 4D-CTA studies, which focused on visual pulsation, raw volumetric changes, or displacement/strain estimation without a formal rule for distinguishing interpretable residual behavior from noise [8,10,13,14,16,40,42].

At the same time, this approach should also be interpreted critically. The residual MAD is not a direct mechanical property of the aneurysm wall but an operational summary of the signal remaining after the subtraction of the dominant periodic component. It may therefore reflect both true non-periodic wall behavior and residual technical variance from segmentation jitter, reconstruction bias, contrast inhomogeneity, phase-dependent noise, or registration errors [15,25,41]. Likewise, the MDC is not a biological cutoff but a repeatability-based interpretive threshold: values below the MDC should be considered not demonstrable above uncertainty, rather than biologically absent. In practical terms, the MAD/MDC framework improves the specificity at the expense of sensitivity. It limits false-positive biological interpretation, but it may also suppress true low-amplitude wall behavior, reduce the effective sample size, and contribute to false-negative associations with growth [15,16,25,26,33,34,41].

### 4.3. Deformability, Variability, Morphology, and Size Change

In the present framework, the first-harmonic pulsation amplitude represents the dominant periodic, pulse-synchronous component of aneurysm motion rather than the entirety of wall behavior. This is biologically plausible because prior in vivo studies showed that aneurysm motion follows the cardiac cycle but is also frequently local, heterogeneous, and sometimes out of phase across sac regions [9,10,12,15,16]. A larger first-harmonic amplitude therefore suggests a stronger repeatable expansion–contraction response of the sac, which may reflect greater dynamic compliance, stronger pulse-wave transmission, or the geometric amplification of cyclic loading. However, this signal must be interpreted cautiously because the measured amplitudes are usually very small and close to the detection limits of current imaging [15,25,41].

By contrast, deformability is more appropriately interpreted as the component of wall behavior that remains after the subtraction of the dominant periodic pulsation and after the exclusion of variation explainable by measurement uncertainty. In signal terms, this corresponds to the residual fluctuation around the first-harmonic fit. Biomechanically, such a residual may reflect non-sinusoidal and spatially heterogeneous wall responses, local delays in deformation, shifting flow patterns, or heterogeneous stiffness across the wall. It should therefore not be viewed as a direct material constant of the aneurysm wall but rather as a conservative descriptor of additional wall motion complexity beyond the main periodic component. This is precisely why using the residual MAD together with the MDC is useful: the residual MAD summarizes the non-coherent remainder of the signal, but only residual variability exceeding uncertainty is interpreted as deformability [26,33,34].

A separate concept is total variability, which should not be conflated with deformability. Total variability reflects the overall observed excursion of a geometric signal across the cardiac cycle before removing the dominant harmonic component. It therefore contains the combined effect of regular pulsation, non-periodic or heterogeneous motion, and residual measurement fluctuation. In practical terms, the total variability asks how much the measured geometry fluctuates overall. The residual MAD-based deformability asks how much fluctuation remains after the dominant periodic motion has been removed and the residual exceeds uncertainty. These descriptors are related but capture different levels of aneurysm dynamics.

This distinction helps to explain our results. In our cohort, the first-harmonic pulsation amplitude did not show a consistent relationship with geometric progression, with only weak-to-moderate and directionally inconsistent correlations across geometric parameters (see Section 3). In contrast, variability showed clearer relationships with morphology: baseline shape irregularity, particularly the undulation index, correlated strongly with surface- and volume-based variability metrics, with a Spearman’s ρ up to ~0.90 (see Section 3). This suggests that an irregular aneurysm geometry generates a more complex dynamic signal but that such complexity should not automatically be equated with growth. Rather, variability may reflect the dynamic complexity of shape behavior, while deformability may reflect the non-periodic fraction of this complexity that survives strict uncertainty filtering. This may also explain why SWP was more clinically interpretable in our cohort than either the global pulsation amplitude or deformability: spatial mapping identifies the locations of mechanically active regions, whereas the latter descriptors summarize the signal more globally.

Recent 4D-CTA studies support this interpretation. Chen et al. found that aneurysms with irregular pulsation showed larger dynamic changes in size (0.59 ± 0.14 mm vs. 0.32 ± 0.12 mm; *p* = 0.010), size% (10.49 ± 1.43% vs. 3.95 ± 1.79%; *p* < 0.001), volume% (13.72% vs. 6.39%; *p* = 0.009), and OSI (0.02 ± 0.01 vs. 0.004 ± 0.005; *p* = 0.004) during the cardiac cycle [37]. Likewise, Xie et al. reported higher stepwise first principal strain in aneurysms with irregular pulsation (0.20 ± 0.01 vs. 0.16 ± 0.02; *p* = 0.033), while the total displacement and total strain did not differ significantly, suggesting that localized or stepwise dynamic descriptors may be more informative than global summaries alone [16]. Together, these findings support a hierarchical interpretation of aneurysm dynamics: the first-harmonic amplitude reflects the dominant periodic motion, total variability reflects the full observed excursion, and deformability reflects the non-periodic remainder beyond the expected uncertainty [16,37].

### 4.4. Relation to Rupture-Focused Pulsation Studies

Most aneurysm pulsation studies have focused on rupture, showing associations between irregular pulsation and ruptured aneurysms, symptomatic aneurysms, or conventional rupture risk factors [38,39,43,44]. These studies support the biological relevance of pulsation, but they are still largely cross-sectional and retrospective [43,44]. For a prospective exploratory study, growth or size change is a more practical and arguably more informative checkpoint, because rupture is rare, ethically difficult to study prospectively, and may itself alter the aneurysm morphology [15,44]. The longitudinal size change, in contrast, reflects structural progression and can be observed in follow-up imaging. This approach is also supported by Hayakawa et al., who reported that, among 20 aneurysms with pulsation, six showed a later shape change, compared with only 2 of 36 aneurysms without pulsation (*p* = 0.04, OR 7.286) [45]. Thus, growth should not be considered only as a surrogate for rupture but also an earlier remodeling event on the same instability continuum [15,42].

### 4.5. Clinical Implications and Limitations

If confirmed in larger prospective cohorts, SWP may provide complementary aneurysm-specific information beyond static morphology and risk scores, particularly in borderline cases, in small aneurysms with uncertain treatment indications, or in patients with multiple aneurysms where treatment prioritization is difficult. However, the present data do not support the use of SWP, GVP, or deformability as standalone treatment markers. At this stage, they should be regarded as exploratory imaging biomarkers that may enrich, but not replace, standard clinical and morphological assessment.

The main limitations of this study include the small single-center cohort, sparse event counts, wide confidence intervals, exploratory statistics without multivariable modeling, threshold dependence, and residual sensitivity to reconstruction, segmentation, image noise, and physiological variability between scans. In addition, all image segmentation preprocessing was performed by a single experienced observer, and formal intraobserver and interobserver variability were not assessed. Although we attempted to reduce segmentation ambiguity by including only aneurysms with a relatively simple adjacent vascular anatomy and excluding cases with extensive adherent contact between the sac and surrounding vessels, some degree of observer dependence cannot be excluded. These limitations are not unique to the present study but reflect broader constraints of the field [15,41,44,46]. Accordingly, our findings should be interpreted primarily as a proof of concept that spatially resolved wall motion assessment may be more informative than the global volumetric change, while conservative deformability analysis provides a useful framework for avoiding the overinterpretation of uncertain signals.

## 5. Conclusions

In this prospective pilot cohort of 11 unruptured intracranial aneurysms in 10 patients, a size change occurred in 6/11 aneurysms (54.5%) during follow-up. Baseline SWP was present in 9/11 aneurysms (82%) and GVP in 8/11 (73%). All aneurysms with a size change showed baseline SWP (6/6), whereas no aneurysm without SWP was enlarged (0/2; *p* = 0.18), while GVP showed no comparable relationship with growth (4/8 vs. 2/3; *p* = 1.00). Variability was associated mainly with baseline shape irregularity, particularly the undulation index (ρ up to ~0.90), rather than with growth. Deformability findings remained preliminary because several metrics were available in fewer than five paired observations after reliability filtering. Given the small sample size and exploratory design, these findings should be interpreted cautiously and require validation in larger, adequately powered longitudinal studies before clinical implementation.

Finally, based on the imaging findings, two patients consented to treatment. The patient with an A3 aneurysm underwent microsurgical treatment, while the patient with an A10 aneurysm received endovascular treatment.

## Figures and Tables

**Table 1:** Patient demographics and clinical characteristics at baseline.

Characteristic	Value
Age, years—mean ± SD	63.1 ± 13.2
Age, years—median (range)	68 (32–79)
Female sex, *n* (%)	5 (45.5)
Male sex, *n* (%)	6 (54.5)
Multiple aneurysms, *n* (%)	7 (63.6)
History of SAH, *n* (%)	1 (9.1)
Smoking, *n* (%)	6 (54.5)
Alcohol consumption, *n* (%)	1 (9.1)
Arterial hypertension, *n* (%)	9 (81.8)
Interval between baseline and follow-up, years (mean ± SD, range)	4.3 ± 1.1 (2–6)

SAH—subarachnoid hemorrhage.

**Table 2:** Patient demographics and aneurysm morphological characteristics at baseline and follow-up.

Patient No.	Age (years)	Gender (M/F)	Aneurysm No.	Location	Size_max_ BL (mm)	Volume BL (mm^3^)	Size_max_ FU (mm)	Volume FU (mm^3^)	FU Interval (years)	ELAPSS 3 y Risk (%)
1	70	F	1	MCA	6.64 ± 0.12	65.05 ± 2.41	6.85 ± 0.09	81.59 ± 1.4	6	17.5
2	79	M	2	AComA	7.49 ± 0.14	87.79 ± 1.96	6.92 ± 0.04	91.72 ± 0.76	3	17.5
3	58	F	3	MCA	4.60 ± 0.07	22.04 ± 0.44	5.19 ± 0.10	27.53 ± 0.72	4	42.7
4	68	M	4	MCA	3.49 ± 0.17	12.41 ± 1.21	3.87 ± 0.12	14.26 ± 0.85	5	11.7
4	68	M	5	PComA	6.71 ± 0.16	58.13 ± 2.02	6.33 ± 0.08	57.56 ± 1.8	5	11.7
5	52	M	6	MCA	4.09 ± 0.10	17.89 ± 0.77	4.42 ± 0.06	23.42 ± 0.49	4	17.5
6	32	M	7	VA	9.80 ± 0.11	203.73 ± 3.87	10.38 ± 0.14	220.85 ± 3.2	5	7.8
7	66	F	8	ICA bifur	4.91 ± 0.09	31.08 ± 0.59	4.64 ± 0.13	31.01 ± 1.27	5	25.8
8	69	F	9	MCA	5.06 ± 0.04	36.36 ± 1.01	5.06 ± 0.09	36.89 ± 1.41	4	17.5
9	51	F	10	BA	7.84 ± 0.20	62.2 ± 2.32	8.95 ± 0.07	164.27 ± 4.2	2	17.5
10	71	M	11	ICA bifur	7.99 ± 0.10	102.07 ± 0.98	8.04 ± 0.12	90.82 ± 0.93	4	11.7

M = male; F = female; BL = baseline; FU = follow-up; MCA = middle cerebral artery; AComA = anterior communicating artery; PComA = posterior communicating artery; VA = vertebral artery; BA = basilar artery; ICA bifur = internal carotid artery bifurcation; y = years. Values for aneurysm size and volume are presented as mean ± measurement variability derived from repeated segmentation. One patient presented with two aneurysms (cases 4 and 5).

**Table 3:** Associations between pulsation type (spatial wall and global volumetric) and observed size change.

Pulsation Type	Size Change	Stable	Total
Spatial wall pulsation			
Present	6	3	9
Absent	0	2	2
Global volumetric pulsation			
Present	4	4	8
Absent	2	1	3

**Table 4:** Association of ELAPSS score, global volumetric pulsation, and spatial wall displacement with aneurysm growth.

ELAPSS Category	Size Change Yes/No	Global Volumetric Pulsation *n*/*N* (%)	Spatial Wall Pulsation *n*/*N* (%)
<20% (*n* = 9)	yes (*N* = 5)	4/5 (80%)	5/5 (100%)
	no (*N* = 4)	4/4 (100%)	3/4 (75%)
≥20% (*n* = 2)	yes (*N* = 1)	0/1 (0%)	1/1 (100%)
	no (*N* = 1)	0/1 (0%)	0/1 (0%)
Aneurysm growth detected		4/6 (67%)	6/6 (100%)

Values are presented as *n*/*N* (%), where *n* represents the number of aneurysms with the specified pulsation pattern and *N* the total number of aneurysms within the corresponding subgroup.

**Table 5:** Associations between pulsation progression and geometric metrics.

Parameter Δ	Spearman ρ	*p*-Value
Linear geometry		
Height	0.109	0.750
Height_max_	−0.491	0.125
Size_max_	0.073	0.832
Neck geometry		
Neck_area_	0.345	0.298
Neck_max_	0.473	0.142
Neck_perimet_	0.291	0.385
Surface		
S_sac_	−0.209	0.537
S_sacpar_	0.391	0.235
Volume		
V_par_	0.264	0.433
V_sac_	−0.255	0.450
V_sacpar_	0.400	0.223

Δ indicates the change between baseline and follow-up measurements. Associations between aneurysm size change and geometric parameters were evaluated using Spearman correlation coefficients (ρ) for 11 paired observations. Bold values indicate the largest observed correlations (|ρ| ≥ 0.40).

**Table 6:** Correlations between ratio-based morphology parameters and baseline geometric measures.

Ratio-Based Morphology Parameter at Baseline	Geometric Measures	Correlation (ρ)	Interpretation
Undulation Index (UI)	S_sac_	0.75–0.85	Moderate–strong
	S_sacpar_	0.85–0.95	Strong
	V_sac_	0.70–0.85	Moderate–strong
	V_sacpar_	0.85–0.95	Strong
	V_par_	≈0.90	Strong
Non-Sphericity Index (NSI)	S_sac_, S_sacpar_, V_sac_, V_sacpar_, V_par_	Weak	Weak associations

Baseline linear and neck-related variables (Height, Height_max_, Size_max_, N_area_, N_max_, N_perimet_) showed no meaningful correlations with ratio-based morphology parameters. AR (aspect ratio), SR (size ratio), BF (bottleneck factor), and CP (conicity parameter) demonstrated no significant associations with the evaluated geometric measures. Correlations were assessed using Spearman coefficients (ρ).

**Figure A1:**
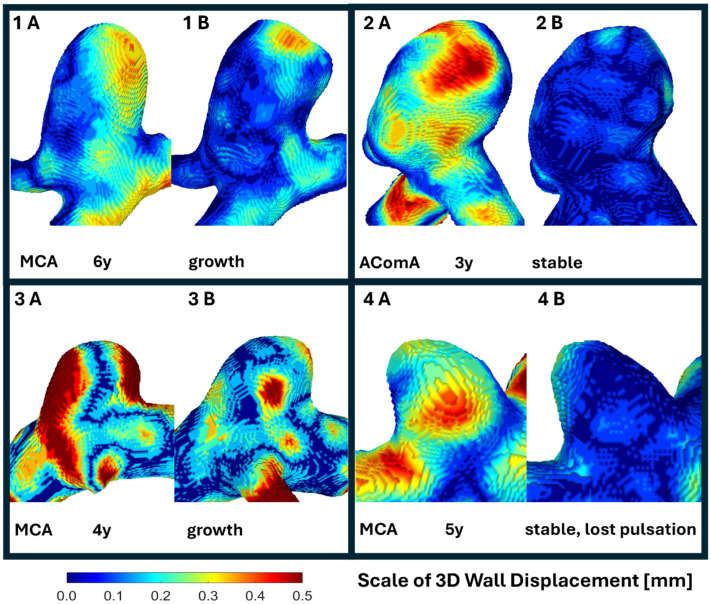
Spatial distribution of aneurysm wall motion during the cardiac cycle in four aneurysms at baseline (**A**) and follow-up (**B**). Color maps show 3D wall displacement (0–0.5 mm), where blue indicates minimal motion and yellow–red indicates greater displacement. MCA—middle cerebral artery; AcomA—anterior communicating artery.

**Figure A2:**
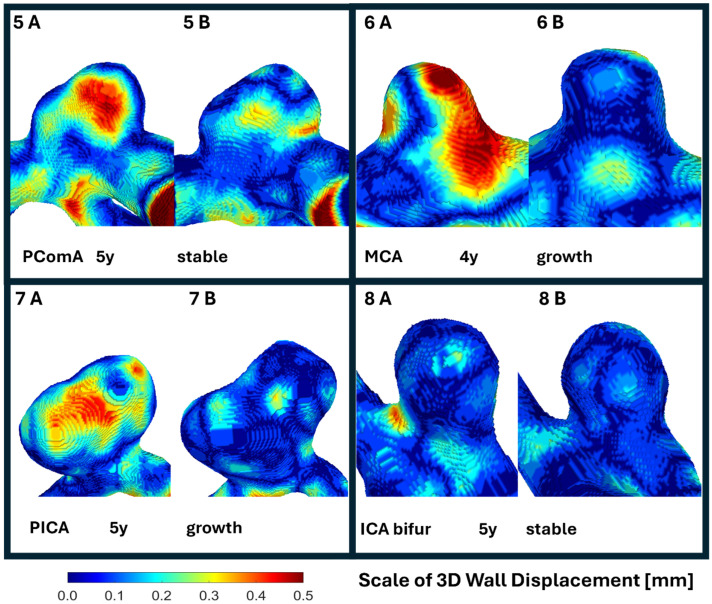
Spatial distribution of aneurysm wall motion during the cardiac cycle in four aneurysms at baseline (**A**) and follow-up (**B**). Color maps show 3D wall displacement (0–0.5 mm), where blue indicates minimal motion and yellow–red indicates greater displacement. PcomA—posterior communicating artery; MCA—middle cerebral artery; PICA—posterior inferior cerebellar artery; ICA bifur – internal carotid artery bifurcation.

**Figure A3:**
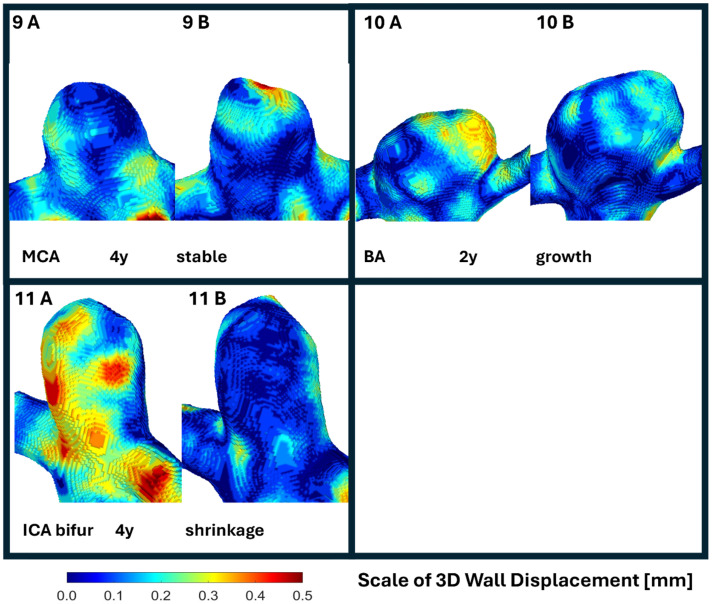
Spatial distribution of aneurysm wall motion during the cardiac cycle in four aneurysms at baseline (**A**) and follow-up (**B**). Color maps show 3D wall displacement (0–0.5 mm), where blue indicates minimal motion and yellow–red indicates greater displacement. MCA—middle cerebral artery; BA—basilar artery; ICA bifur—internal carotid artery bifurcation.

## Data Availability

Data available on demand.

## Supplementary Materials

The following supporting information can be downloaded at https://www.mdpi.com/article/10.3390/neurolint18050081/s1. Table S1: List of the geometric parameters, abbreviations, and descriptions; Table S2: Baseline demographic, clinical, and aneurysm characteristics (*N* = 11); Table S3: Patient-level ELAPSS scores, pulsation characteristics, and size change outcomes; Table S4: Full Spearman correlation statistics between baseline morphology and deformability metrics.

## Appendix A

### Appendix A.1. Signal Differentiation Using First Harmonic Amplitude, Residual MAD, and Repeatability Thresholds

Phase-resolved geometric measurements obtained from ECG-gated 4D-CTA represent a mixture of periodic cardiac pulsation, non-periodic wall deformation, and measurement noise [15,16,25,41]. To separate these components, the geometric signal sampled across the cardiac cycle (20 phases) was decomposed into periodic and residual components.

### Appendix A.2. Harmonic Modelling of the Periodic Component

The periodic component of the signal was modeled using a first-order harmonic fit, representing the fundamental oscillation driven by the cardiac cycle [30,31]:

$$X_{fit}(t)=a_{0}+a_{1}cos(\omega t)+b_{1}sin(\omega t)$$

where

- X(t) is the geometric measurement at cardiac phase t
- $a_{0}$ is the mean value of the signal
- $a_{1}$ and $b_{1}$ are harmonic coefficients
- ω is the angular frequency of the cardiac cycle.

The amplitude of the first harmonic

$$A_{1}=\sqrt{a_{1}^{2}+b_{1}^{2}}$$

was used to quantify the magnitude of pressure-driven pulsation [30,31].

A first-harmonic model was intentionally used instead of full Fourier decomposition because aneurysm wall motion during the cardiac cycle is dominated by the fundamental pressure waveform, while higher-order components in 4D-CTA signals are highly sensitive to segmentation noise, reconstruction artefacts, and phase jitter [15,25,41].

### Appendix A.3. Extraction of the Residual Signal

After estimating the periodic component, it was subtracted from the original signal to obtain residuals:

$$r_{i}=X_{i}-X_{fit,i}$$

where $X_{i}$ represents the measured geometric value at phase i.

These residuals contain both measurement uncertainty and potential non-periodic biological deformation.

### Appendix A.4. Quantification of Residual Variability

Residual variability was quantified using the median absolute deviation (*MAD*) [26,27]:

$$MAD=\mathrm{median}\left(|r_{i}-\mathrm{median}\left(r\right)|\right)$$

*MAD* was selected as a robust estimator of dispersion that is less sensitive to outliers and non-Gaussian noise than variance-based measures such as standard deviation [26,27].

Residual *MAD* therefore represents the overall magnitude of non-periodic variability remaining after removal of the periodic pulsation component.

### Appendix A.5. Repeatability Analysis and Minimum Detectable Change

To distinguish true biological variability from measurement uncertainty, repeatability was evaluated using Bland–Altman analysis [32,33].

For paired measurements obtained under identical conditions, the difference between measurements was calculated as

$$d_{i}=X_{1,i}-X_{2,i}$$

The mean difference represents measurement bias:

$$\bar{d}=\frac{1}{n}\sum d_{i}$$

and the standard deviation of the differences is

$$SD_{d}$$

The 95% limits of agreement (*LOA*) were calculated [32,33] as

$$LOA=\bar{d}\pm 1.96\cdot SD_{d}$$

These limits define the expected range of measurement variability due to acquisition and segmentation uncertainty.

Based on the repeatability analysis, the minimum detectable change (*MDC*) [33,34,35] was calculated as

$$MDC=1.96\cdot \sqrt{2}\cdot SD_{d}$$

The *MDC* represents the smallest change that exceeds measurement error with 95% confidence [34,35].

## Appendix B

**Case 1:** Focal pulsation on the right side of the sac at baseline. At 6-year follow-up, aneurysm growth and visually detectable morphological change occur at the initial pulsation site, with loss of detectable pulsation.

**Case 2:** Heterogeneous pulsation at baseline. Stable morphology and pulsation pattern at 3-year follow-up without aneurysm growth or visible shape change.

**Case 3:** Heterogeneous pulsation at baseline. Minor aneurysm growth and development of a subtle right-sided sac prominence during the 4-year follow-up, followed by stabilization.

**Case 4:** Focal pulsation at baseline with stable morphology and pulsation pattern during the 5-year follow-up.

**Case 5.** Focal pulsation at baseline disappears during the 5-year follow-up, accompanied by aneurysm stabilization.

**Case 6:** Heterogeneous pulsation at baseline. At 4-year follow-up, aneurysm growth and subtle morphological change are observed, with loss of detectable pulsation.

**Case 7:** Focal pulsation at baseline is followed by aneurysm growth and morphological change over 5 years, with subsequent stabilization of wall motion. The initial pulsation corresponds to the region of the most pronounced morphological transformation.

**Case 8:** No detectable pulsation at baseline and no aneurysm growth or morphological change during follow-up.

**Case 9:** No detectable pulsation at baseline or at the 4-year follow-up. No aneurysm growth is observed, with only subtle morphological remodeling.

**Case 10:** Focal pulsation on the right side of the sac at baseline. At 2-year follow-up, marked aneurysm expansion is observed with loss of detectable pulsation.

**Case 11:** Diffuse heterogeneous pulsation involving most of the sac surface at baseline. At 4-year follow-up, pulsation is no longer detectable, with aneurysm stabilization and mild size reduction.

## References

[1] Etminan N., Brown R.D., Beseoglu K., Juvela S., Raymond J., Morita A., Torner J.C., Derdeyn C.P., Raabe A., Mocco J. (2015). The Unruptured Intracranial Aneurysm Treatment Score: A Multidisciplinary Consensus. Neurology.

[2] Vlak M.H., Algra A., Brandenburg R., Rinkel G.J. (2011). Prevalence of Unruptured Intracranial Aneurysms, with Emphasis on Sex, Age, Comorbidity, Country, and Time Period: A Systematic Review and Meta-Analysis. Lancet Neurol..

[3] Etminan N., Rinkel G.J. (2016). Unruptured Intracranial Aneurysms: Development, Rupture and Preventive Management. Nat. Rev. Neurol..

[4] Investigators U.J., Morita A., Kirino T., Hashi K., Aoki N., Fukuhara S., Hashimoto N., Nakayama T., Sakai M., Teramoto A. (2012). The Natural Course of Unruptured Cerebral Aneurysms in a Japanese Cohort. N. Engl. J. Med..

[5] Greving J.P., Wermer M.J.H., Brown R.D., Morita A., Juvela S., Yonekura M., Ishibashi T., Torner J.C., Nakayama T., Rinkel G.J.E. (2014). Development of the PHASES Score for Prediction of Risk of Rupture of Intracranial Aneurysms: A Pooled Analysis of Six Prospective Cohort Studies. Lancet Neurol..

[6] Al-Khindi T., Macdonald R.L., Schweizer T.A. (2010). Cognitive and Functional Outcome After Aneurysmal Subarachnoid Hemorrhage. Stroke.

[7] van Gijn J., Kerr R.S., Rinkel G.J. (2007). Subarachnoid Haemorrhage. Lancet.

[8] Kato Y., Hayakawa M., Sano H., Sunil M., Imizu S., Yoneda M., Watanabe S., Abe M., Kanno T. (2004). Prediction of Impending Rupture in Aneurysms Using 4D-CTA: Histopathological Verification of a Real-Time Minimally Invasive Tool in Unruptured Aneurysms. Min-Minim. Invasive Neurosurg..

[9] Hayakawa M., Katada K., Anno H., Imizu S., Hayashi J., Irie K., Negoro M., Kato Y., Kanno T., Sano H. (2005). CT Angiography with Electrocardiographically Gated Reconstruction for Visualizing Pulsation of Intracranial Aneurysms: Identification of Aneurysmal Protuberance Presumably Associated with Wall Thinning. Am. J. Neuroradiol..

[10] Ishida F., Ogawa H., Simizu T., Kojima T., Taki W. (2005). Congress of Neurological Surgeons/American Association of Neurological Surgeons Joint Section Chairmen. Neurosurgery.

[11] Matsumoto M., Sasaki T., Suzuki K., Sakuma J., Endo Y., Kodama N. (2006). Visualizing the Dynamics of Cerebral Aneurysms with Four-Dimensional Computed Tomographic Angiography. Neurosurgery.

[12] Karmonik C., Diaz O., Grossman R., Klucznik R. (2009). In-Vivo Quantification of Wall Motion in Cerebral Aneurysms from 2D Cine Phase Contrast Magnetic Resonance Images. RöFo Fortschritte Auf Dem Geb. Röntgenstrahlen Bild. Verfahr..

[13] Kuroda J., Kinoshita M., Tanaka H., Nishida T., Nakamura H., Watanabe Y., Tomiyama N., Fujinaka T., Yoshimine T. (2012). Cardiac Cycle-Related Volume Change in Unruptured Cerebral Aneurysms. Stroke.

[14] Dissaux B., Ognard J., Aouni M.C.E., Nonent M., Haioun K., Magro E., Gentric J.C. (2020). Volume Variation May Be a Relevant Metric in the Study of Aneurysm Pulsatility: A Study Using ECG-Gated 4D-CTA (PULSAN). J. NeuroInterv. Surg..

[15] Stam L.B., Aquarius R., de Jong G.A., Slump C.H., Meijer F.J.A., Boogaarts H.D. (2021). A Review on Imaging Techniques and Quantitative Measurements for Dynamic Imaging of Cerebral Aneurysm Pulsations. Sci. Rep..

[16] Xie H., Yu H., Wu H., Wang J., Wu S., Zhang J., Zhao H., Yuan M., Mendieta J.B., Anbananthan H. (2024). Quantifying Irregular Pulsation of Intracranial Aneurysms Using 4D-CTA. J. Biomech..

[17] Fedorov A., Beichel R., Kalpathy-Cramer J., Finet J., Fillion-Robin J.-C., Pujol S., Bauer C., Jennings D., Fennessy F., Sonka M. (2012). 3D Slicer as an Image Computing Platform for the Quantitative Imaging Network. Magn. Reson. Imaging.

[18] Koizumi S., Kin T., Shono N., Kiyofuji S., Umekawa M., Sato K., Saito N. (2024). Patient-Specific Cerebral 3D Vessel Model Reconstruction Using Deep Learning. Med. Biol. Eng. Comput..

[19] Hsu W.-C., Meuschke M., Frangi A.F., Preim B., Lawonn K. (2025). A Survey of Intracranial Aneurysm Detection and Segmentation. Med. Image Anal..

[20] Kottner J., Audigé L., Brorson S., Donner A., Gajewski B.J., Hróbjartsson A., Roberts C., Shoukri M., Streiner D.L. (2011). Guidelines for Reporting Reliability and Agreement Studies (GRRAS) Were Proposed. J. Clin. Epidemiol..

[21] Dhar S., Tremmel M., Mocco J., Kim M., Yamamoto J., Siddiqui A.H., Hopkins L.N., Meng H. (2008). Morphology parameters for intracranial aneurysm rupture risk assessment. Neurosurgery.

[22] Raghavan M.L., Ma B., Harbaugh R.E. (2005). Quantified Aneurysm Shape and Rupture Risk. J. Neurosurg..

[23] Dhar R., Diringer M.N. (2015). Relationship between Angiographic Vasospasm, Cerebral Blood Flow, and Cerebral Infarction after Subarachnoid Hemorrhage. Acta Neurochir. Suppl..

[24] Kleinloog R., Zwanenburg J.J.M., Schermers B., Krikken E., Ruigrok Y.M., Luijten P.R., Visser F., Regli L., Rinkel G.J.E., Verweij B.H. (2018). Quantification of Intracranial Aneurysm Volume Pulsation with 7T MRI. Am. J. Neuroradiol..

[25] Linden S.M.L., Stam L.B., Aquarius R., Hering A., de Korte C.L., Prokop M., Boogaarts H.D., Meijer F.J.A., Oostveen L.J. (2024). Feasibility of Capturing Vessel Expansion with 4D-CTA: Phantom Study to Determine Reproducibility, Spatial and Temporal Resolution. Med. Phys..

[26] Rousseeuw P.J., Croux C. (1993). Alternatives to the Median Absolute Deviation. J. Am. Stat. Assoc..

[27] Leys C., Ley C., Klein O., Bernard P., Licata L. (2013). Detecting Outliers: Do Not Use Standard Deviation around the Mean, Use Absolute Deviation around the Median. J. Exp. Soc. Psychol..

[28] Shidhore T.C., Cohen-Gadol A.A., Rayz V.L., Christov I.C. (2022). Comparative Assessment of Biomechanical Parameters in Subjects with Multiple Cerebral Aneurysms Using Fluid–Structure Interaction Simulations. J. Biomech. Eng..

[29] Backes D., Rinkel G.J.E., Greving J.P., Velthuis B.K., Murayama Y., Takao H., Ishibashi T., Igase M., terBrugge K.G., Agid R. (2017). ELAPSS Score for Prediction of Risk of Growth of Unruptured Intracranial Aneurysms. Neurology.

[30] Chen J., Faber T.L., Cooke C.D., Garcia E.V. (2008). Temporal Resolution of Multiharmonic Phase Analysis of ECG-Gated Myocardial Perfusion SPECT Studies. J. Nucl. Cardiol..

[31] Atchley A.E., Trimble M.A., Samad Z., Shaw L.K., Pagnanelli R., Chen J., Garcia E.V., Iskandrian A.E., Velazquez E.J., Borges-Neto S. (2009). Use of Phase Analysis of Gated SPECT Perfusion Imaging to Quantify Dyssynchrony in Patients with Mild-to-Moderate Left Ventricular Dysfunction. J. Nucl. Cardiol..

[32] Giavarina D. (2015). Understanding Bland Altman Analysis. Biochem. Med..

[33] Bland J.M., Altman D.G. (2010). Statistical Methods for Assessing Agreement between Two Methods of Clinical Measurement. Int. J. Nurs. Stud..

[34] de Vet H.C.W., Terwee C.B., Knol D.L., Bouter L.M. (2006). When to Use Agreement versus Reliability Measures. J. Clin. Epidemiol..

[35] Lee P., Liu C.-H., Fan C.-W., Lu C.-P., Lu W.-S., Hsieh C.-L. (2013). The Test–Retest Reliability and the Minimal Detectable Change of the Purdue Pegboard Test in Schizophrenia. J. Formos. Med. Assoc..

[36] Holmes D.T., Buhr K.A. (2007). Error Propagation in Calculated Ratios. Clin. Biochem..

[37] Chen S., Zhang W., Cheng Y., Wang G., Lv N. (2024). Quantification of Morpho-Hemodynamic Changes in Unruptured Intracranial Aneurysms with Irregular Pulsation during the Cardiac Cycle Using 4D-CTA. Front. Neurol..

[38] Zhou J., Guo Q., Chen Y., Lin B., Ding S., Zhao H., Pan Y., Wan J., Zhao B. (2022). Irregular Pulsation of Intracranial Aneurysm Detected by Four-Dimensional CT Angiography and Associated with Small Aneurysm Rupture: A Single-Center Prospective Analysis. Front. Neurol..

[39] Zhang J., Li X., Zhao B., Zhang J., Sun B., Wang L., Ding S., Liu X., Yan J., Mossa-Basha M. (2021). Irregular Pulsation of Intracranial Unruptured Aneurysm Detected by Four-Dimensional CT Angiography Is Associated with Increased Estimated Rupture Risk and Conventional Risk Factors. J. NeuroInterv. Surg..

[40] Firouzian A., Manniesing R., Metz C.T., Risselada R., Klein S., van Kooten F., Sturkenboom M.C.J.M., van der Lugt A., Niessen W.J. (2013). Quantification of Intracranial Aneurysm Morphodynamics from ECG-Gated CT Angiography. Acad. Radiol..

[41] Schetelig D., Sedlacik J., Fiehler J., Frölich A., Knopp T., Sothmann T., Waschkewitz J., Werner R. (2018). Analysis of the Influence of Imaging-Related Uncertainties on Cerebral Aneurysm Deformation Quantification Using a No-Deformation Physical Flow Phantom. Sci. Rep..

[42] Hayakawa M., Tanaka T., Sadato A., Adachi K., Ito K., Hattori N., Omi T., Oheda M., Katada K., Murayama K. (2014). Detection of Pulsation in Unruptured Cerebral Aneurysms by ECG-Gated 3D-CT Angiography (4D-CTA) with 320-Row Area Detector CT (ADCT) and Follow-up Evaluation Results: Assessment Based on Heart Rate at the Time of Scanning. Clin. Neuroradiol..

[43] Zhang J., Li X., Zhao B., Zhang J., Sun B., Wang L., Tian J., Mossa-Basha M., Kim L.J., Yan J. (2023). Irregular Pulsation of Aneurysmal Wall Is Associated with Symptomatic and Ruptured Intracranial Aneurysms. J. NeuroInterv. Surg..

[44] Vanrossomme A.E., Eker O.F., Thiran J.-P., Courbebaisse G.P., Boudjeltia K.Z. (2015). Intracranial Aneurysms: Wall Motion Analysis for Prediction of Rupture. Am. J. Neuroradiol..

[45] Hayakawa M., Maeda S., Sadato A., Tanaka T., Kaito T., Hattori N., Ganaha T., Moriya S., Katada K., Murayama K. (2011). Detection of Pulsation in Ruptured and Unruptured Cerebral Aneurysms by Electrocardiographically Gated 3-Dimensional Computed Tomographic Angiography With a 320-Row Area Detector Computed Tomography and Evaluation of Its Clinical Usefulness. Neurosurgery.

[46] Stam L.B., Linden S.M.L., Oostveen L.J., Hansen H.H.G., Aquarius R., Slump C.H., de Korte C.L., Bartels R.H.M.A., Prokop M., Boogaarts H.D. (2023). Dynamic Computed Tomography Angiography for Capturing Vessel Wall Motion: A Phantom Study for Optimal Image Reconstruction. PLoS ONE.

