# Supplementary material for: ECG-Gated 4D-CTA Assessment of Intracranial Aneurysm Wall Dynamics and Longitudinal Size Change: An Exploratory Study
Source: Neurol Int. 2026 Apr 27;18(5):81. doi: 10.3390/neurolint18050081 (PMC13210108; doi:10.3390/neurolint18050081)
Supplement: Supplementary file 1 [file neurolint-18-00081-s001.zip › neurolint-4220070-supplementary.pdf]

Supplementary Table S1

List of the geometric parameters, abbreviation and description

**Aneurysm sac**		
Height, mm	H	maximal perpendicular distance from neck cut plane to the aneurysm surface [22]
Maximum height	H_{max}	Maximum distance between centroid of the neck and the aneurysm surface [21]
Maximum size	L_{max}	Maximum distance between two points on the aneurysm surface
Volume of the sac	V_{sac}	Volume of the aneurysm above anatomic neck
Volume of the sac + parent artery	V_{sacpar}	Volume of the aneurysm with adjacent parent arteries, which is cut D1 at the proximal neck and D2 at 1,5* D1 upstream
Volume of the parent artery	V_{par}	Volume of adjacent parent arteries, is counted as V_{sacpar} - V_{sac}
Surface of the sac	S_{sac}	Surface of the aneurysm above the anatomic neck
Surface of the sac + parent artery	S_{sacpar}	Surface of the aneurysm with adjacent parent arteries, which is cut D1 at the proximal neck and D2 at 1,5* D1 upstream
Convex hull volume	V_{ch}	Is the smalest volume that fully encloses the IA volume above the neck
**Neck**		
Maximum neck diameter	N_{max}	Maximum distance between two points on the neck [22]
Area of the anatomical neck	N_{area}	Area af the anatomical neck [22]
Perimeter of the neck	$N_{perimet}$	Perimeter of the neck [22]
**Ratio-based parameter**		
Aspect ratio		Ratio of the height to maximum neck diameter [21]
Size ratio		Ratio of maximum aneurysm height/ avarege vessel diameter<br>(D1 at the proximal neck and D2 at 1,5X D1 upstream) [22]
Undulation index	UI	1- (V_{sac}/V_{ch}) [22]
Non-sphericity index	NSI	$1 - (18\pi)^{1/3} V^{2/3} / S_{sac}$ [22]
Bootle neck factor	BF	BF= D_{max}/ D_n, $D_n = 4*N_{area}/ N_{perimeter}$ [22]
Conicity parameter	CP	0,5-(H_b/H), H_b -is the height of the cross-sectionof the sac from the neck plane at which D_{max} occurs [22]

Supplementary Table S2

Baseline Demographic, Clinical, and Aneurysm Characteristics (*N* = 11)

Characteristic	Value
Age, years – Mean ± SD	63.1 ± 13.2
Age, years – Median (range)	68 (32–79)
Female sex, *n* (%)	5 (45.5)
Male sex, *n* (%)	6 (54.5)
Multiple aneurysms, *n* (%)	7 (63.6)
History of SAH, *n* (%)	1 (9.1)
Aneurysm localisation	
– MCA	4 (36.4)
– ICA bifurcation	3 (27.3)
– PComA	2 (18.2)
– Basilar artery	1 (9.1)
– VA–PICA	1 (9.1)
Smoking, *n* (%)	6 (54.5)
Alcohol consumption, *n* (%)	1 (9.1)
Arterial hypertension, *n* (%)	9 (81.8)
Diabetes mellitus, *n* (%)	4 (36.4)
Ischaemic NCMP, *n* (%)	3 (27.3)
Family history of IA and/or SAH, *n* (%)	2 (18.2)
Family history of bleeding without aneurysm, *n* (%)	0 (0)
Unruptured aneurysm in family members, *n* (%)	3 (27.3)
Indication for cerebral vessel examination	
– Ischaemic stroke	3 (27.3)
– SAH from different aneurysm	1 (9.1)
– Visual disturbance / amaurosis	2 (18.2)
– Tinnitus	1 (9.1)
– Syncope	1 (9.1)
– Head trauma	1 (9.1)
– Cefalea	1 (9.1)
– Vertigo	1 (9.1)

Values are presented as mean ± SD, median (range), or number (percentage).

Abbreviations: MCA – middle cerebral artery; ICA – internal carotid artery; PComA – posterior communicating artery; BA – basilar artery; PICA – posterior inferior cerebellar artery; SAH – subarachnoid hemorrhage.

Supplementary Table S3

Patient-Level ELAPSS Score, Pulsation Characteristics, and Size Change Outcome

Patient	ELAPSS score, 3-years risk, (%)	Baseline volumetric pulsation	Baseline spatial pulsation (3D colour map)	Size change	Follow-up volumetric pulsation	Follow-up spatial pulsation
P1	17.5	Pulsation	Local pulsation	Yes	Pulsation	Non-pulsation
P2	17.5	Pulsation	Heterogenic pulsation	No	Non-pulsation	Non-pulsation
P3	42.7	Non-pulsation	Heterogenic pulsation	Yes	Non-pulsation	Non-pulsation
P4	11.7	Pulsation	Local pulsation	No	Non-pulsation	Non-pulsation
P5	11.7	Pulsation	Local pulsation	No	Pulsation	Non-pulsation
P6	17.5	Non-pulsation	Heterogenic pulsation	Yes	Non-pulsation	Non-pulsation
P7	7.8	Pulsation	Local pulsation	Yes	Non-pulsation	Non-pulsation
P8	25.8	Non-pulsation	Non-pulsation	No	Pulsation	Non-pulsation
P9	17.5	Pulsation	Non-pulsation	No	Pulsation	Non-pulsation
P10	17.5	Pulsation	Local pulsation	Yes	Pulsation	Non-pulsation
P11	11.7	Pulsation	Heterogenic pulsation	Yes	Pulsation	Non-pulsation

Supplementary Table S4.

Full Spearman correlation statistics between baseline morphology and deformability metrics

Spearman correlation coefficient (ρ), nominal p-value, and FDR-corrected q-value
(n = 11 aneurysms)

Morphologic index	***Deformability metric***	***ρ (Spearman)***	***p-value***	***FDR q-value***	***Interpretation***
UI	*Ssac*	*0.75–0.85*	*< 0.01*	*< 0.05*	*Significant*
UI	*Ssacpar*	*0.85–0.95*	*< 0.001*	*< 0.01*	*Significant*
UI	*Vsac*	*0.70–0.85*	*< 0.01*	*< 0.05*	*Significant*
UI	*Vsacpar*	*0.85–0.95*	*< 0.001*	*< 0.01*	*Significant*
UI	*Vpar*	*≈ 0.90*	*< 0.001*	*< 0.01*	*Significant*
NSI	*Ssac*	*~0.40–0.55*	*0.05–0.10*	*> 0.10*	*Trend*
NSI	*Ssacpar*	*~0.40–0.55*	*0.05–0.10*	*> 0.10*	*Trend*
NSI	*Vsac*	*~0.40–0.55*	*0.05–0.10*	*> 0.10*	*Trend*
NSI	*Vsacpar*	*~0.40–0.55*	*0.05–0.10*	*> 0.10*	*Trend*
NSI	*Vpar*	*~0.40–0.55*	*0.05–0.10*	*> 0.10*	*Trend*

Height	*All deformability metrics*	*< 0.30*	*> 0.10*	*> 0.10*	*Not significant*
Height_max	*All deformability metrics*	*< 0.30*	*> 0.10*	*> 0.10*	*Not significant*
Size_max	*All deformability metrics*	*< 0.30*	*> 0.10*	*> 0.10*	*Not significant*
N_area	*All deformability metrics*	*< 0.30*	*> 0.10*	*> 0.10*	*Not significant*
N_perimeter	*All deformability metrics*	*< 0.30*	*> 0.10*	*> 0.10*	*Not significant*
N_max	*All deformability metrics*	*< 0.30*	*> 0.10*	*> 0.10*	*Not significant*
AR	*All deformability metrics*	*< 0.25*	*> 0.10*	*> 0.10*	*Not significant*
SR	*All deformability metrics*	*< 0.25*	*> 0.10*	*> 0.10*	*Not significant*
BF	*All deformability metrics*	*< 0.25*	*> 0.10*	*> 0.10*	*Not significant*
CP	*All deformability metrics*	*< 0.25*	*> 0.10*	*> 0.10*	*Not significant*
